# Machine learning prediction of prostate cancer from transrectal ultrasound video clips

**DOI:** 10.3389/fonc.2022.948662

**Published:** 2022-08-26

**Authors:** Kai Wang, Peizhe Chen, Bojian Feng, Jing Tu, Zhengbiao Hu, Maoliang Zhang, Jie Yang, Ying Zhan, Jincao Yao, Dong Xu

**Affiliations:** ^1^ Department of Ultrasound, The Affiliated Dongyang Hospital of Wenzhou Medical University, Dongyang, China; ^2^ College of Optical Science and Engineering, Zhejiang University, Hangzhou, China; ^3^ Department of Ultrasound, Cancer Hospital of the University of Chinese Academy of Sciences, Zhejiang Cancer Hospital, Hangzhou, China; ^4^ Institute of Basic Medicine and Cancer (IBMC), Chinese Academy of Sciences, Hangzhou, China; ^5^ Key Laboratory of Head & Neck Cancer Translational Research of Zhejiang Province, Hangzhou, China; ^6^ Zhejiang Provincial Research Center for Cancer Intelligent Diagnosis and Molecular Technology, Hangzhou, China

**Keywords:** artificial intelligence, prostate cancer, ultrasound, machine learning, support vector machine

## Abstract

**Objective:**

To build a machine learning (ML) prediction model for prostate cancer (PCa) from transrectal ultrasound video clips of the whole prostate gland, diagnostic performance was compared with magnetic resonance imaging (MRI).

**Methods:**

We systematically collated data from 501 patients—276 with prostate cancer and 225 with benign lesions. From a final selection of 231 patients (118 with prostate cancer and 113 with benign lesions), we randomly chose 170 for the purpose of training and validating a machine learning model, while using the remaining 61 to test a derived model. We extracted 851 features from ultrasound video clips. After dimensionality reduction with the least absolute shrinkage and selection operator (LASSO) regression, 14 features were finally selected and the support vector machine (SVM) and random forest (RF) algorithms were used to establish radiomics models based on those features. In addition, we creatively proposed a machine learning models aided diagnosis algorithm (MLAD) composed of SVM, RF, and radiologists’ diagnosis based on MRI to evaluate the performance of ML models in computer-aided diagnosis (CAD). We evaluated the area under the curve (AUC) as well as the sensitivity, specificity, and precision of the ML models and radiologists’ diagnosis based on MRI by employing receiver operator characteristic curve (ROC) analysis.

**Results:**

The AUC, sensitivity, specificity, and precision of the SVM in the diagnosis of PCa in the validation set and the test set were 0.78, 63%, 80%; 0.75, 65%, and 67%, respectively. Additionally, the SVM model was found to be superior to senior radiologists’ (SR, more than 10 years of experience) diagnosis based on MRI (AUC, 0.78 vs. 0.75 in the validation set and 0.75 vs. 0.72 in the test set), and the difference was statistically significant (*p*< 0.05).

**Conclusion:**

The prediction model constructed by the ML algorithm has good diagnostic efficiency for prostate cancer. The SVM model’s diagnostic efficiency is superior to that of MRI, as it has a more focused application value. Overall, these prediction models can aid radiologists in making better diagnoses.

## Introduction

Prostate cancer (PCa) is one of the most common cancers in males, and its prevalence has increased at an alarming rate over the last several decades (1). According to GLOBOCAN 2020, in 2020 there were approximately 1,414,259 new cases of PCa and 375,304 PCa-related deaths worldwide, with a particularly high prevalence in developed countries (2). The early clinical manifestations of prostate cancer are sufficiently nonspecific that patients often ignore it in its early phases and therefore only seek treatment when it has already developed. Therefore, early diagnosis of PCa is crucial. Prostate-specific antigen (PSA) testing, digital rectal examinations (DRE), and transrectal ultrasonography (TRUS) guided prostate system biopsies are the most used PCa screening methods in clinics (3, 4), but these diagnostic tools may still lead to a certain degree of overdiagnosis (5).

In the past decade, the role of MRI in the diagnosis of prostate cancer stages has significantly developed. The introduction of coil imaging in the rectum and the advent of some basic techniques, such as magnetic resonance spectrum imaging, dynamic contrast-enhanced MRI, and diffusion-weighted imaging (DWI), have improved the diagnostic accuracy of MRI and its potential to improve the treatment decision-making process (6). However, it must be emphasized that multiparametric magnetic resonance imaging (mpMRI) has been evaluated only in patients in whom the risk of clinically significant PCa was judged sufficiently high to warrant biopsy. Therefore, a prebiopsy mpMRI must not be used as an initial screening tool. Indeed, based on its low specificity, mpMRI in very low-risk patients would result in an increase in false-positive findings and subsequent unnecessary biopsies (5). Another classical imaging diagnosis of PCa has largely relied on ultrasonography (US), including transrectal ultrasound (TRUS), contrast-enhanced ultrasonography (7), and ultrasound elastography (8). However, sonologists’ evaluations of tumor tissue have primarily relied on semantic features from the visual perspective, which is an approach that misses many image features that represent tumor heterogeneity. Therefore, early accurate diagnosis of prostate cancer remains a clinical challenge.

As a new frontier, ML-based radiomics could extract many quantitative features from encrypted digital images, which could then be used to deeply mine the biological information of tumors and analyze the heterogeneity of tumors, thus aiding clinical decision making (9). However, it has been reported that the ML ultrasound diagnostic model is rarely used to evaluate PCa because prostate cancers located in the central zone are often difficult to detect visually—they are confused with the hypoechoic endoglandular background tissue. Plus, the application of ML methods on prostate cancer prediction is mostly based on static transrectal images, which cannot fully display the patient's tissue information, meaning that the application of ML methods for prostate cancer prediction based on transrectal video clips remains problematic.

Accordingly, we adopted ML models based on whole prostate transrectal ultrasound video clips on PCa prediction. The ML algorithms were better at forming predictions because they could use the ultrasound video clips to make a prediction based on global information from whole prostate transrectal tissue. To test the performance of the ML algorithms, we compared their diagnostic results with radiologists’ diagnosis based on MRI, finding that the SVM algorithm adopted in this paper had better performance in terms of PCa prediction. In addition, we creatively proposed a machine learning models aided diagnosis algorithm (MLAD) to evaluate the performance of ML models in computer-aided diagnosis (CAD).

## Materials and methods

Local ethics committees approved the study (2022-YX-047).

### Study participants

We obtained the ultrasound video clips data from Dongyang Hospital, which is affiliated with Wenzhou Medical University. From January 2021 to December 2021, we recruited 276 patients with PCa and 225 patients with benign lesions of the prostate, which included benign prostatic hyperplasia, fibromuscular tissue, atypical glandular tissue, and chronic prostatitis.

The inclusion criteria were as follows: (a) was an elderly male (aged above 55); (b) had solid prostate masses found by digital rectal examination, TRUS, or MRI; (c) had undergone prostate biopsy or surgery and obtained the pathological diagnosis results; and (d) had not received treatment for prostate diseases before TRUS. In addition, we excluded patients with rectal malformation or rectal surgery who could not be examined by transrectal ultrasound.

**Figure 1:**
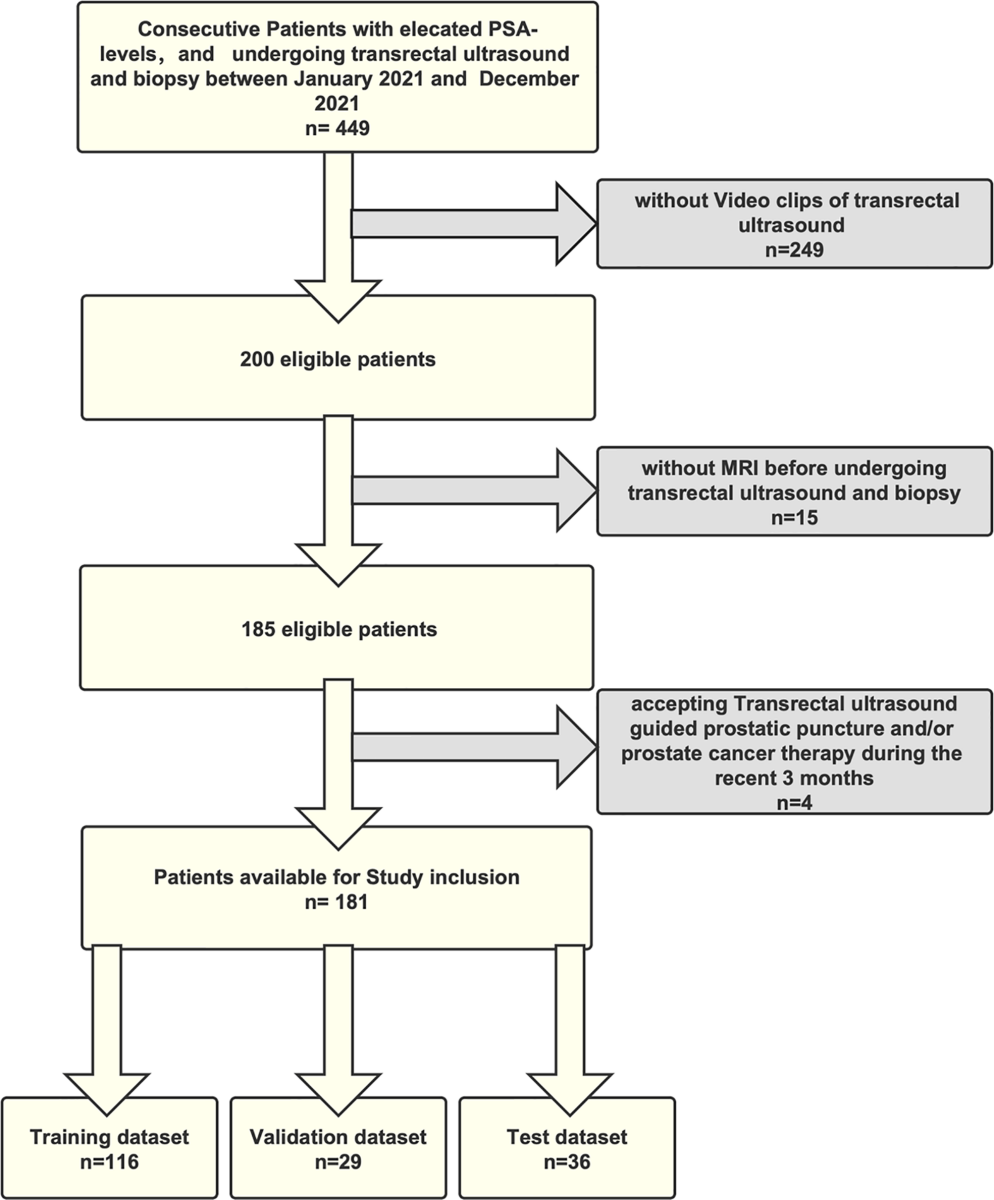
The flowchart of inclusion and exclusion of the study population.

Finally, of the 231 patients—113 having PCa and 118 benign lesions—we randomly selected 170 for the purpose of training and validating an ML model, while using the remaining 61 to test derived models. The processes underlying the inclusion and exclusion of study participants are shown in **Figure 1**.

### Video clips acquisition

We collected all ultrasound data related to the prostate using the Esaote MyLab™ ClassC ultrasound machine (Esaote, Genoa, Italy) with the TRT33 Transrectal Biplane Transducer (frequency range 3–13MHz). Four sonologists, each with over ten years of experience in transrectal ultrasound, performed all ultrasound scans. First, they placed a condom on the TRT33 probe, then inserted it into the rectum, adjusted the probe depth, and rotated the probe for multidirectional prostate examination. Second, they scanned the entire transverse section of the prostate grayscale ultrasound, before scanning the prostate from top to bottom and storing 10 seconds of video clips.

### Manual segmentation

For manual segmentation, we loaded the video clips into the 3D Slicer v.4.11. Two sonologists (S1 and S2, each with more than five years of TRUS prostate diagnosis experience) manually segmented the region of interest (ROI) from the prostate. They were blinded to the MRI and pathological results. We drew the entire prostate gland as an ROI from every video frame and used intra- and inter-class correlation coefficients (ICCs) to evaluate the reproducibility of radiomics feature extraction. First, S1 and S2 separately segmented video clips of 30 randomly selected patients, and then, two weeks later, S2 segmented images of the 30 patients once more. After that, S1 performed the remaining video clip segmentation. We only included the features with an ICC value equal to or higher than 0.8 that indicated excellent reproducibility in the other feature selection process.

### Feature extraction and selection

We performed extractions of radiomics features by using a radiomics extension of a 3D Slicer software, SlicerRadomics (Version 3.0.1) (10). For this, we extracted 851 radiomics features from each patient, including shape features (14), first-order statistical features (18), gray-level co-occurrence matrix features (24), gray-level dependence matrix features (14), gray-level run-length matrix features (16), gray-level size zone matrix features (16), neighborhood gray-tone difference matrix features (5), and wavelet-based features (744).

We performed feature selection using programs written in Python (Version 3.8.8, Python Software Foundation). First, according to pathological results, we divided all data into benign and malignant groups and inserted labels 0 and 1 into the data. Second, we used the two independent samples t-test and Mann-Whitney U test to test all the features, before deleting the features in the benign and malignant groups that failed to meet either of the first two tests. Third, the least absolute shrinkage and selection operator (LASSO) regression selected features in the training and validation set. We excluded the features with zero variance using the variance filtering method. Fourth, we performed the LASSO method for further dimensionality reduction of the features and selected the most valuable features (11). We then repeated the 10-fold cross-validation on training and validation set process 100,000 times to obtain the optimal value of parameter λ, which we introduced into the LASSO method to calculate the regression coefficients of each feature. Finally, we selected the features with non-zero coefficients.

### Machine learning

Python scikit-learn 0.24.2 package (12) was used to support vector machine modeling and evaluation. We randomly divided the training and validation set into the training set and the validation set at a ratio of 8:2. First, we used a Gaussian kernel support vector machine (SVM) model and a random forest (RF) model to classify features in the training set and established two nonlinear classifiers. In the SVM classifier, kernel size parameters (γ, gamma) and regularization parameters (C, cost) of the SVM kernel function were optimized. We then selected the parameters with the best performance through 10-fold cross-validation on the training set. In the RF classifier, the number of estimators (n_estimator) was optimized through 10-fold cross-validation on the training set. Finally, we applied the SVM model and RF model to the validation set and test set.

### MRI results collection

Participants underwent prostate MRIs with a 1.5T Siemens Magnetom Avanto magnetic resonance scanner (Erlangen, Germany), including the standard T2-weighted MRI, T1-weighted MRI, and diffusion-weighted MRI. We included the MRI results of the validation and test set in the analysis. Two junior radiologists (each with less than 5 years of experience) and two senior radiologists (each with more than 10 years of experience) reviewed each case in order to provide an independent diagnosis.

### Machine learning models aided diagnosis algorithm

Using the SVM model and RF model, we can obtain the probabilities of PCa separately. To evaluate the performance of ML models in CAD, we proposed a machine learning models aided diagnosis algorithm (MLAD) (Equation 1) which integrates the prediction performance of SVM, RF, and radiologists’ diagnosis. In this algorithm, we chose SVM as the main model and RF as the sub model.


(1) $$\{\begin{array}{c} S_{SVM}=\;\left|0.5-P_{SVM}\right|\\ S_{RF}=\;\left|0.5-P_{RF}\right|\\ S_{MLAD}=\;\frac{S_{SVM}}{0.5}*P_{SVM}+\;\frac{0.5-S_{SVM}}{0.5}*\;\left(\frac{S_{RF}}{0.5}*P_{RF}+\;\frac{0.5-S_{RF}\;}{0.5}*V_{R}\right)\; \end{array}$$

*P_SVM_*: probability of PCa from SVM model; *P_RF_*: probability of PCa from RF model; *V_R_*: result value of PCa from radiologists’ diagnosis (0: benign, 1: malignant); *S_SVM_*: prediction confidence score of SVM; *S_RF_*: prediction confidence score of RF

### Statistical analysis

**Figure 2:**
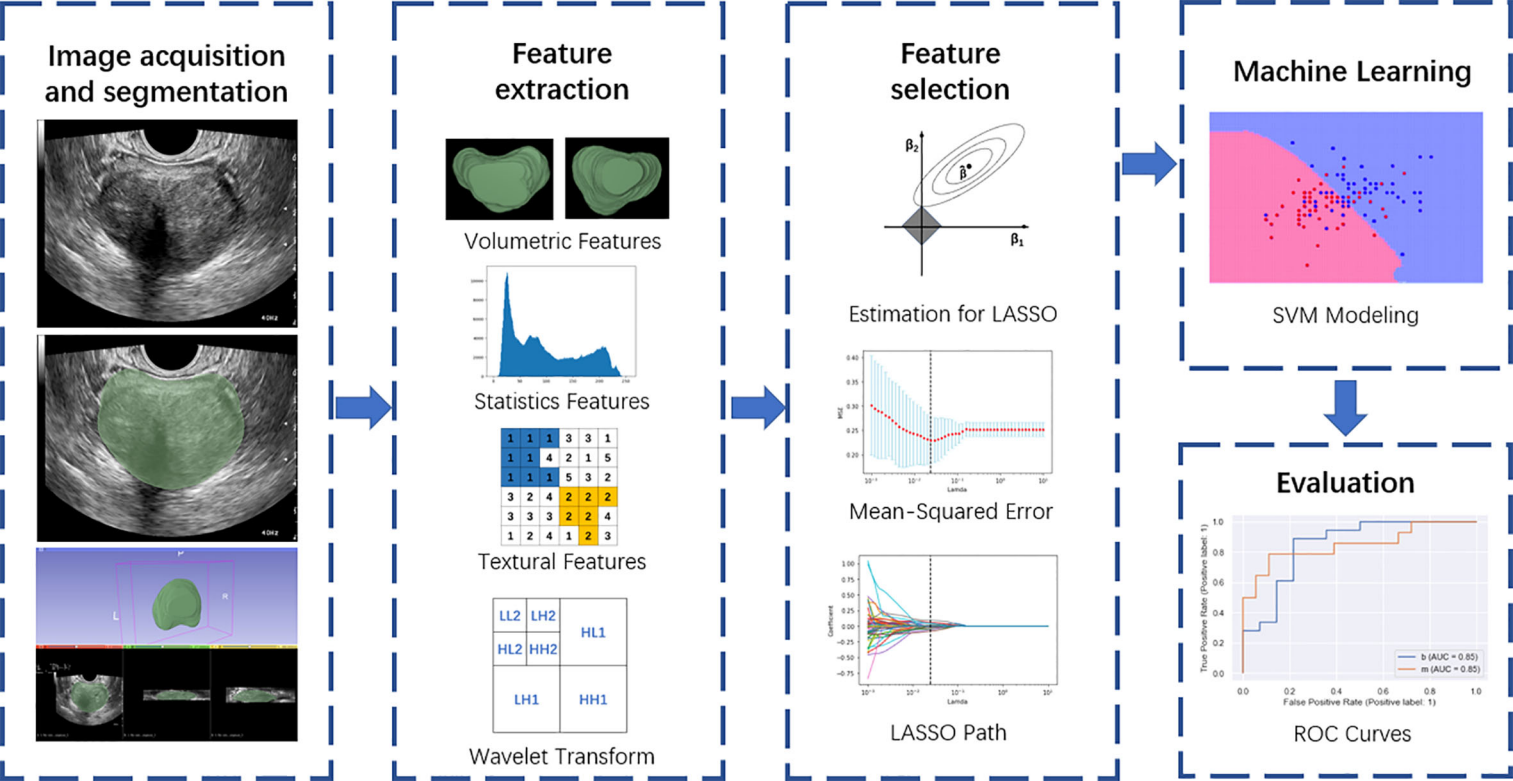
Overall flow chart of the study, including image acquisition and segmentation, feature extraction, feature selection, machine learning, and evaluation.

We used SPSS 25.0 software to conduct a statistical analysis and tested the normality of continuous variables using the Levene test. We analyzed continuous variables obeying a normal distribution by using the independent samples t-test and analyzed those not obeying a normal distribution by using the Mann-Whitney U test. We then compared categorical variables using the chi-square test. Unless otherwise specified, we expressed the continuous variables as median (standard deviation, SD) and the categorical variables as n (%). We also evaluated the area under the curve (AUC) as well as the sensitivity, specificity, and precision of the SVM model and MRI in diagnosing prostate cancer by employing receiver operator characteristic curve (ROC) analysis. *P*< 0.05 indicated a significant difference. The overall flowchart of the study is outlined in **Figure 2**.

## Results

### Clinicopathological features of the patients

The clinicopathological features in the training set, validation set, and test set are shown in **Table 1**. The average ages of patients in the training set, validation set, and test set were 72.02, 71.21, and 69.64, respectively, while the respective mean PSA values were 19.91, 22.77, and 46.98. The number of benign lesions were 83 (48.8%), 15 (44.1%), and 30 (49.2%). The number of PCa were 87 (51.2%), 19 (55.9%), and 31 (50.8%). We found no significant differences between the validation and test set in terms of age, PSA, or pathological results (*p* > 0.05).

**Table 1:** Characteristics of patients in the training, validation and test datasets.

	Training set	Validation set	Test set	*P* value
Age(y)*	72.02±8.721	71.21±6.246	69.64±8.262	0.161
PSA(ng/mL)*	19.91±44.56	22.77±65.51	46.98±114.89	0.065
Pathology				0.871
No.of Benign(-)(%)	83(48.8%)	15(44.1%)	30(49.2%)	
BPH	68(40%)	13(38.3%)	25(41%)	
BPH & prostatitis	11(6.4%)	1(2.9%)	3(4.9%)	
BPH & BCH	1(0.6%)	0	2(3.2)	
BPH & LGIN	3(1.8%)	1(2.9%)	0	
No. of Pca(+)(%)	87(51.2%)	19(55.9%)	31(50.8%)	
GS6	30(17,6%)	9(26.5%)	14(23%)	
GS7	38(22.4%)	7(20.6%)	8(13.1%)	
GS8	10(5.9%)	2(5.9%)	6(9.8%)	
GS>=8	9(5.3%)	1(2.9%)	3(4.9%)	

BPH, benign prostatic hyperplasia; BCH, basal cell hyperplasia; LGIN. low-grade intraepithelial neoplasia.*Data are expressed as mean ± standard deviation.p< 0.05 indicates significant differences in patients’ clinicopathological features in the validation and test sets.

### Feature selection

From each patient, we extracted 851 features from the ultrasound video clips using the LASSO regression model in the training set. In the LASSO model, we repeated the 10-fold cross-validation process 100,000 times in order to generate the optimal penalization coefficient lambda (λ).

**Figure 3:**
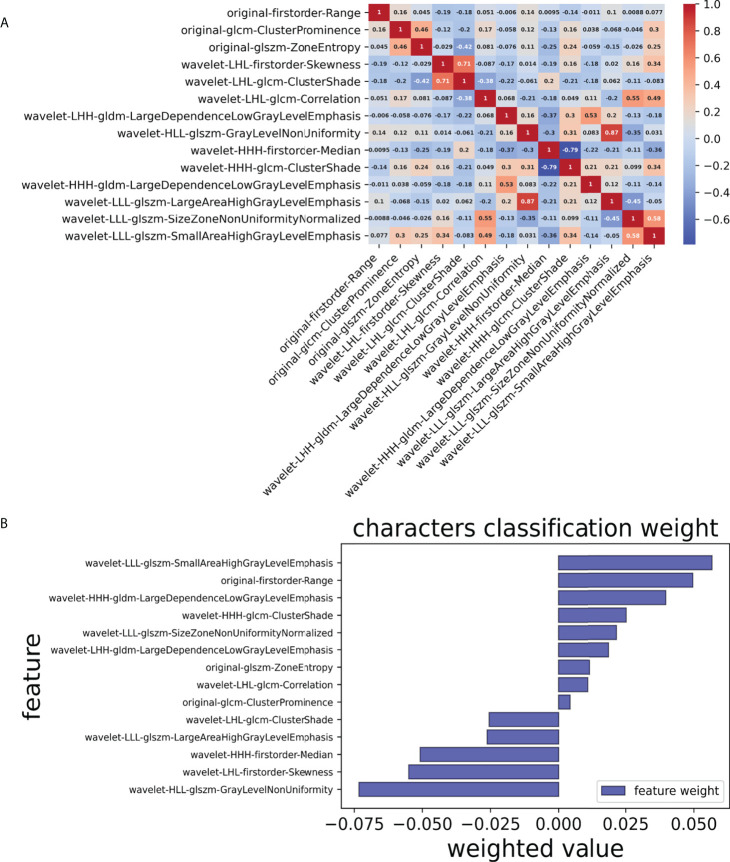
Selection of significant parameters in features in the training set and definition of the linear predictor. **(A)** Spearman’s correlation coefficients were calculated for the fourteen selected features. **(B)** Characters classification weight of the features.

**Figure 4:**
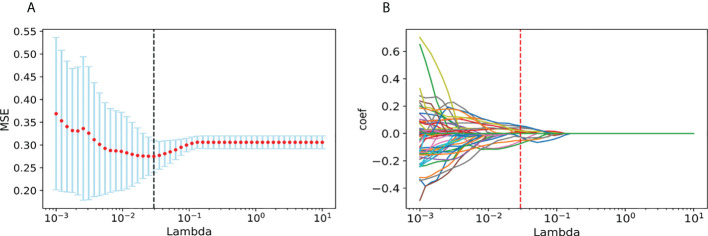
Generation of the optimal penalization coefficient lambda. **(A)** Ten-time cross-validation for tuning parameter selection in the LASSO model. **(B)** LASSO coefficient solution path for the 14 features.

Finally, we chose a λ value of 0.029470517025518096. After dimensionality reduction with LASSO regression, 14 features were selected, consisting of original (3) and wavelet features (11). The subset of features ultimately selected by the LASSO algorithm is shown in **Table 2**. **Figure 3**, meanwhile, shows the selection of significant parameters in features in the training set and the definition of the linear predictor, while **Figure 4** shows the generation of the optimal penalization coefficient lambda.

**Table 2:** The subset of radiomics features ultimately selected by the LASSO algorithm.

Feature	Image type	Feature Class	Feature Name	LASSO coefficients
1	Original	Firstorder	Range	0.049561
2	Original	glcm	ClusterProminence	0.004193
3	Original	glszm	ZoneEntropy	0.011709
4	Wavelet-LHL	firstorder	Skewness	-0.055034
5	Wavelet-LHL	glcm	ClusterShade	-0.025479
6	Wavelet-LHL	glcm	Correlation	0.010685
7	Wavelet-LHH	gldm	LargeDependenceLowGrayLevelEmphasis	0.018646
8	Wavelet-HLL	glszm	GrayLevelNonUniformity	-0.073279
9	Wavelet-HHH	firstorder	Median	-0.050800
10	Wavelet-HHH	glcm	ClusterShade	0.025124
11	Wavelet-HHH	gldm	LargeDependenceLowGrayLevelEmphasis	0.039665
12	Wavelet-LLL	glszm	LargeAreaHighGrayLevelEmphasis	-0.026230
13	Wavelet-LLL	glszm	SizeZoneNonUniformityNormalized	0.021580
14	Wavelet-LLL	glszm	SmallAreaHighGrayLevelEmphasis	0.056598

First-order features describe the distribution of voxel intensities within the image region defined by the mask through commonly used and basic metrics. GLCM features describe the second-order joint probability function of an image region constrained by the mask. They are defined as P. GLDM features quantify gray-level dependencies in an image, and GLSZM features quantify gray-level zones in an image.

### Modeling and effectiveness

**Figure 5:**
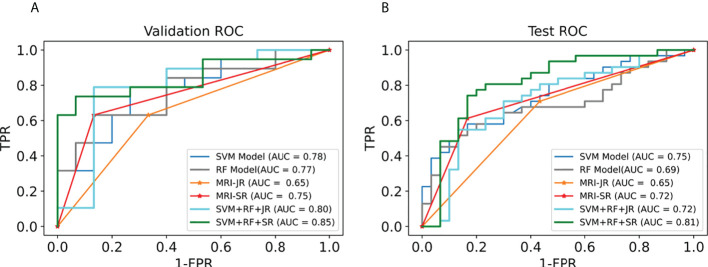
Comparison of ROC between the ML models and MRI in the validation set and test set. **(A)** shows the ROC curves of the validation set. **(B)** shows the ROC curves of the test set. (MRI-JR: junior radiologists’ diagnosis based on MRI, MRI-SR: senior radiologists’ diagnosis based on MRI).

We first selected the SVM algorithm to establish the radiomics model based on the selected 14 features. Traditionally, the prediction performance has been optimized for the following parameters: C, gamma, and the shape of the kernel. We then constructed a pipeline with two steps: a scaling step and an SVM step. It is best to scale data before passing them to an SVM. Next, we varied the relevant RBF parameters, C and gamma, logarithmically, varying by one order of magnitude at a time. We used a 10-fold cross-validation scheme. Finally, we identified the best SVM estimator (C=45.20, gamma=0.001) and stored it as an SVM model. As with the SVM model, in the RF algorithm, we varied the number of estimators by one order of magnitude at a time in order to obtain the best RF model (n_estimators=10000). The AUC, sensitivity, specificity, and precision of the SVM and RF model in the diagnosis of PCa in the validation set and the test set were as follows: (1) SVM results: 0.78, 63% (95%CI: 0.38–0.83), 80% (95%CI: 0.51–0.95), 80% (0.51–0.95); 0.75, 65% (95%CI: 0.45–0.80), 67% (95%CI: 0.47–0.82), 67% (95%CI: 0.47–0.82); (2) RF results: 0.77, 63% (95%CI: 0.39–0.83), 87% (95%CI: 0.58–0.98), 86% (95%CI: 0.56–0.97); 0.69, 45% (95%CI: 0.28–0.64), 93% (95%CI: 0.76–0.99), 88% (95%CI: 0.60–0.98). For comparison, two junior radiologists (each with less than 5 years of experience) and two senior radiologists (each with more than 10 years of experience) respectively gave their independent diagnosis of PCa based on MRI results. The AUC, sensitivity, specificity, and precision of two radiologists in the diagnosis of PCa based on MRI were as follows: (1) JR results: 0.65, 63% (95%CI: 0.39–0.83), 67% (95%CI: 0.39–0.87), 71% (95%CI: 0.44–0.87); 0.65, 71% (95%CI: 0.52–0.85), 60% (95%CI: 0.41–0.77), 65% (95%CI: 0.46–0.80); (2) SR results: 0.75, 63% (95%CI: 0.39–0.83), 87% (95%CI: 0.58–0.98), 86% (95%CI: 0.56–0.97); 0.72, 61% (95%CI: 0.42–0.78), 83% (95%CI: 0.65–0.93), 0.79 (95%CI: 0.57–0.92) (**Table 3**). According to the statistical results, the SVM model was superior to radiologists’ diagnosis based on MRI (AUC, 0.78 vs. 0.65/0.75 and 0.75 vs. 0.65/0.72) (**Figure 5**), and the results of the SVM model and SR were statistically significant (*p*< 0.05). To evaluate the performance of ML models in CAD, we integrated the SVM model and RF model with JR and SR diagnosis through MLAD separately (SVM+RF+JR and SVM+RF+SR). The AUC, sensitivity, specificity, and precision of the MLAD model in the diagnosis of PCa in the validation set and the test set were as follows: (1) SVM+RF+JR: 0.8, 74% (95%CI: 0.49–0.90), 87% (95%CI: 0.58–0.98), 88% (95%CI: 0.60–0.98); 0.72, 68% (95%CI: 0.49, 0.83), 70% (95%CI: 0.50, 0.85), 70% (95%CI: 0.50, 0.85); (2) SVM+RF+SR: 0.86, 74% (95%CI: 0.49–0.90), 93% (95%CI: 0.66–0.99), 93% (95%CI: 0.66–0.99); 0.81, 81% (95%CI: 0.62, 0.92), 80% (95%CI: 0.61–0.92), 81% (95%CI: 0.62–0.92). According to the statistical results, the MLAD model with senior radiologists’ diagnosis (SVM+RF+SR) was superior to senior radiologists’ diagnosis based on MRI and the SVM model (AUC, 0.85 vs. 0.75/0.78 and 0.81 vs. 0.72/0.75) (**Figure 5**), and the results were statistically significant (*p*< 0.05). The results thus demonstrated that the SVM model and RF model can improve the predictive performance of PCa through MLAD.

**Table 3:** Diagnostic performance of machine learning model and MRI on a per-lesion basis.

Dataset and Method	Sensitivity (95% CI)	Specificity (95% CI)	Precision (95% CI)	AUC	*P* Value	Kappa
**SVM Model**						
**Validation**	0.63 (0.38-0.83)	0.80 (0.51-0.95)	0.80 (0.51-0.95)	0.78	0.012	0.42
**Test**	0.65 (0.45-0.80)	0.67 (0.47-0.82)	0.67 (0.47-0.82)	0.75	0.015	0.312
**RF Model**						
**Validation**	0.63 (0.37-0.83)	0.87 (0.58-0.98)	0.86 (0.56-0.97)	0.77	0.003	0.481
**Test**	0.45 (0.28-0.64)	0.93 (0.76-0.99)	0.88 (0.60-0.98)	0.69	0.001	0.382
**MRI-JR**						
**Validation**	0.63 (0.39-0.83)	0.67 (0.39-0.87)	0.71 (0.44-0.87)	0.65	0.084	0.294
**Test**	0.71 (0.52-0.85)	0.60 (0.41-0.77)	0.65 (0.46-0.80)	0.65	0.015	0.31
**MRI-SR**						
**Validation**	0.63 (0.39-0.83)	0.87 (0.58-0.98)	0.86 (0.56-0.97)	0.75	0.003	0.481
**Test**	0.61 (0.42-0.78)	0.83 (0.65-0.93)	0.79 (0.57-0.92)	0.72	0.0003	0.445
**SVM+RF+JR**						
**Validation**	0.74 (0.49-0.90)	0.87 (0.58-0.98)	0.88 (0.60-0.98)	0.8	0.000464	0.591
**Test**	0.68 (0.49-0.83)	0.70 (0.50-0.85)	0.70 (0.50-0.85)	0.72	0.003	0.377
**SVM+RF+SR**						
**Validation**	0.74 (0.49-0.90)	0.93 (0.66-0.99)	0.93 (0.66-0.99)	0.85	0.00009	0.652
**Test**	0.81 (0.62-0.92)	0.80 (0.61-0.92)	0.81 (0.62-0.92)	0.81	0.000002	0.606

SVM model, support vector machine model; RF model, random forest model; MRI-JR, junior radiologists’ (less than 5 years of experience) diagnosis based on MRI; MRI-SR, senior radiologists’ (more than 5 years of experience) diagnosis based on MRI. p< 0.05 indicates a significant difference in the discrimination of the SVM model and MRI diagnosis.

## Discussion

ML-based radiomics transforms visual image information into in-depth feature quantitative data, extracts a large amount of image characteristic information from medical images, and constructs pre-measurement models based on feature information (13, 14). In this study, we carried out feature extraction of the prostate creatively from ultrasound video clips. The prediction model constructed by the ML algorithm has good diagnostic efficiency in PCa, and, compared with the SVM model with an MRI, the diagnostic efficiency is better and has a more specific application value.

TRUS is widely used in clinical practice because it is safe, radiation-free, inexpensive, and easy to perform (15, 16). The outline of the prostate is usually clearly displayed, and the boundary between the isoechoic peripheral and hypoechoic central zone is demarcated. Approximately 70% of PCa is located in the peripheral area, and most PCa is hypoechoic. However, tumors in the central location are often difficult to detect because they are confused with the low-echoic endoglandular background tissue. Thus, TRUS has only moderate accuracy in PCa detection in the general population (17). Therefore, we used the video clips of the prostate to serve as the feature extraction data in order to avoid losing a key portion of the information.

By extracting high-throughput data and establishing an efficient and stable prediction model, radiomics can provide an auxiliary diagnosis for clinical practice. Features selection is the key to ML research. However, data redundancy and over-fitting will occur if the high-throughput feature extraction is not selected (18–20). Jin et al. used the ML method to predict lymph node metastasis of early cervical cancer and extracted 106 imaging omics features from lymph node ultrasound images. Through a combination of LASSO and ridge regression, they selected the key features from the high-dimensional features to avoid overfitting. They then selected six features for classification research, which represented the texture complexity of tumors and correlated with the high degree of tumor heterogeneity (21). By contrast, in our study, we extracted a total of 851 features. We used the LASSO algorithm to filter all the features and retained only the 14 non-zero features with a solid correlation with PCa. The results show that the AUC of the prediction model in the PCa of the training set, validation set, and test set were respectively 0.82, 0.78, and 0.75. The difference was not statistically significant (*p* >0.05) and it has been shown that the feature selection method used in this study can effectively restrain data overfitting.

Out of 14 selected image omics features (22), one was taken from the first-order range, i.e., the range of gray values in the ROI; one was from the gray-level co-occurrence matrix (GLCM), which describes the second-order joint probability function of an image region constrained by the mask; one was from the gray-level size zone matrix (GLSZM), which quantifies gray level zones in an image; and the remaining 11 were taken from wavelet (23), subsets of texture features. Among the selected radiomics features, texture features based on wavelet account for the majority, which indicates that texture features have a good classification function. Furthermore, they are related to the composition of heterogeneous cells with noticeable molecular and microenvironmental differences in malignant tumors, indicating that the texture characteristics of tumors are highly correlated with heterogeneity (21).

The commonly used modeling methods of radiomics are mainly divided into statistical and ML-based methods. ML approaches are then typically subdivided into supervised and unsupervised learning, with SVM and RF being the most widely used approaches in supervised learning (24–28). Previous literature has reported that both SVM and RF show good stability. Specifically, SVM and RF show good diagnostic efficiency in constructing a small sample prediction model (29). The basic principle of SVM is to divide a hyperplane into a given training queue space to distinguish different types of samples (30). In this study, we used SVM and RF to construct a radiomics prediction model. The AUC of SVM in the validation and test set was 0.78 and 0.75, respectively, showing that the model had high diagnostic efficiency and stability.

To evaluate the diagnostic efficacy of the ML prediction SVM model in PCa, we compared the SVM model with MRI diagnosis. In Rosenkrantz et al.’s study, it was reported that the sensitivity, specificity, and precision of PCa detection using a fusion of T2-weighted images and diffusion-weighted images were 60.8%, 80.3%, and 71.0%, respectively (31). The results of Katahira et al.’s study, meanwhile, which used T2WI and DWI to detect prostate cancer, showed that the sensitivity, specificity, and AUC were 61.2%, 82.6%, and 0.755, respectively (32). In our study, the AUC, sensitivity, specificity, and precision of two radiologists in the diagnosis of PCa based on MRI were 0.65/0.75, 63%/63%, 67%/87%, 71%/86%; 0.65/0.72, 71%/61%, 60%/83%, 65%/79% (the former is the diagnosis results of JR, and the latter is the diagnosis results of SR). The diagnostic power of MRIs in this study was similar to that observed in previous studies. The results show that the SVM model had higher diagnostic efficiency than a diagnosis based on MRI (AUC, 0.78 vs. 0.65/0.75 and 0.75 vs. 0.65/0.72). In the extended experiment, the MLAD model with SR diagnosis (SVM+RF+SR) showed the best performance in statistics, which means the SVM model and RF model can improve radiologists’ diagnosis performance.

In spite of its findings, it must be acknowledged that this study suffers from some limitations. The most obvious limitation is that it was a retrospective study with a small sample size, limiting its power and precluding firm statistical conclusions. For example, the CI of AUC sensitivity, specificity, and precision has a large range. The second limitation is that it was a single-center study, and thus we cannot exclude single-centered effects. Finally, we carried out ROI segmentation manually, which is inefficient and may lead to bias among different delineators, thus resulting in the reduced diagnostic capability of the prediction model. Although the radiomics model performed well in this study, future studies must combine clinical factors closely related to PCa to build a more robust model.

It is rare to use the ML ultrasound diagnostic model to evaluate PCa. Therefore, we aimed to build an ultrasound diagnostic prediction model based on ML to provide a solid theoretical basis for the accurate and individualized treatment of PCa.

## Conclusions

In our study, we innovatively used ultrasound video clips instead of images to form a dataset on which we could build an ML model. The ML-based prediction models have good diagnostic efficiency in PCa. In the SVM model, the precision, sensitivity, and specificity are better than that seen in diagnosis based on MRI. Thus, based on our MLAD, the SVM model and RF model can contribute to improve radiologists’ diagnosis performance based on MRI; indeed, the MLAD model in conjunction with senior radiologists’ diagnosis shows the best performance among all models. In our future work, we intend to combine the ultrasound transverse and longitudinal video clips of the prostate to build a better ML model and use deep learning and neural networks in the ultrasonic diagnosis of prostate cancer. The model developed in our study could contribute to reducing barriers and providing a convenient way for community hospitals to improve PCa diagnosis.

## Data availability statement

The raw data supporting the conclusions of this article will be made available by the authors, without undue reservation.

## Ethics statement

The studies involving human participants were reviewed and approved by Medical Ethics Committee of Dongyang People’s Hospital. The patients/participants provided their written informed consent to participate in this study. Written informed consent was obtained from the individual(s) for the publication of any potentially identifiable images or data included in this article.

## Author contributions

KW and DX designed the study. KW, JT, ZH, MZ, and ZW contributed to data acquisition. PC, JY and YZ carried out statistical analysis. PC, BF, JY, JT, and ZH interpreted the results. KW and JY wrote the draft. PC and BF reviewed and edited the manuscript. All authors read and approved the final version of the manuscript.

## Funding

The study was supported in part by the National Natural Science Foundation of China (82071946), the Natural Science Foundation of Zhejiang Province (LZY21F030001 and LSD19H180001), the Medical and Health Research Project of Zhejiang Province (2021KY099 and 2022KY110) and the funds from the University Cancer Foundation *via* the Sister Institution Network Fund at the University of Texas MD Anderson Cancer Center, and the Key Science and Technology Project of Jinhua, Zhejiang Province (2022-3-017).

## Conflict of interest

The authors declare that the research was conducted in the absence of any commercial or financial relationships that could be construed as a potential conflict of interest.

## Publisher’s note

All claims expressed in this article are solely those of the authors and do not necessarily represent those of their affiliated organizations, or those of the publisher, the editors and the reviewers. Any product that may be evaluated in this article, or claim that may be made by its manufacturer, is not guaranteed or endorsed by the publisher.

## Abbreviations

ML, machine learning; PCa, prostate cancer; MRI, magnetic resonance imaging; SVM, support vector machine; RF, random forest; MLAD, machine learning models aided diagnosis algorithm; CAD, computer-aided diagnosis; JR, junior radiologist; SR, senior radiologist; LASSO, the least absolute shrinkage and selection operator; DWI, diffusion-weighted imaging; AUC, area under the curve; ROC, receiver operator characteristic curve; US, ultrasonography; TRUS, transrectal ultrasound; ICCs, intra- and inter-class correlation coefficients; ROI, region of interest.
